# COVID-19: The Many Ways to Hurt Your Heart

**DOI:** 10.3390/v15020416

**Published:** 2023-02-01

**Authors:** Aklima Akter, Xavier Clemente-Casares

**Affiliations:** 1 Department of Medical Microbiology and Immunology, Faculty of Medicine and Dentistry, University of Alberta, Edmonton, AB T6G 2R3, Canada; 2 Li Ka Shing Institute of Virology, University of Alberta, Edmonton, AB T6G 2E1, Canada; 3 Cardiovascular Research Institute, University of Alberta, Edmonton, AB T6G 1C9, Canada

## 1. Introduction

Coronavirus disease 2019 (COVID-19), caused by the Severe Acute Respiratory Syndrome Coronavirus 2 (SARS-CoV-2), has become a global pandemic, affecting the lives of billions of individuals. Although the virus primarily affects the lungs and causes mild influenza-like illness to Acute Respiratory Distress Syndrome (ARDS), severe SARS-CoV-2 infection can also cause off-target damage, particularly in the heart and vasculature. People with pre-existing cardiovascular diseases (CVDs) have increased susceptibility and high mortality risk with SARS-CoV-2 infections. Furthermore, patients who have recovered from acute myocardial injury may develop cardiac complications after SARS-CoV-2 infection. The several manuscripts published in this Special Issue on “COVID-19-Associated Myocarditis and Cardiac Pathology” address different forms of COVID-19-mediated cardiac pathology. A general review on the topic by Dmytrenko et al. assessed the epidemiology and pathophysiology of cardiovascular sequelae of COVID-19 and proposed a disease mechanism and directed some possible unanswered questions and future directions regarding cardiac manifestations of COVID-19 [1].

### 1.1. SARS-CoV-2 and Myocarditis

Most of the COVID-19-related myocarditis cases in clinical samples have been reported based on indirect cardiac tests [2], such as increased troponin level, diffuse ST-segment elevation on the electrocardiogram, diffuse biventricular hypokinesis on cardiac MRI [3], enlarged heart, and LV dysfunction by echocardiography [4]. Histopathological studies after SARS-CoV-2 infection have also revealed diffuse lymphocyte, monocyte, and neutrophil infiltrates, apparent interstitial edema, and limited focal necrosis [5,6,7]. Additionally, endomyocardial biopsy with immunological light microscopy has revealed large, vacuolated CD68^+^ macrophages with membrane damage and cytoplasmic vacuoles in a patient with COVID-19-related myocarditis [8]. Several animal models and in vitro systems have also been used to improve our understanding of COVID-19 infection and the mechanism of pathogenesis in the heart [9,10,11]. COVID-19 infection of transgenic K18-hACE2 mice is associated with moderate levels of viral RNA in the heart [12,13], with 22% of the heart showing scattered hyper-eosinophilic cardiomyocytes with pyknotic nuclei [13]. Additionally, SARS-CoV-2 infections in a Syrian golden hamster’s model showed focal lymphocytic myocarditis confirmed with CD3+ T lymphocyte staining [14]. In vitro studies using human cell-based heart models, such as cardiac tissue derived from human-induced pluripotent stem cells, have also been developed to understand the direct route of viral entry and pathogenesis and offer an opportunity to study clinically relevant cardiac viral infections [15,16].

In this Issue, Ho et al. reviewed the mechanisms of SARS-CoV-2-induced myocarditis. The authors explain the effects of direct SARS-CoV-2 infections on the heart and how they lead to myocarditis. They also summarize the various mechanisms by which SARS-CoV-2 manipulates host cell biology and outline potential treatment options [17].

Overall, COVID-19-associated myocarditis has proven to be a rare phenomenon, both in humans and in animal models. However, COVID-19 patients who recover from viral myocarditis present more subtle residual myocardial changes, mainly low-grade inflammation and fibrosis [18], which can lead to long-term cardiac complications [19,20,21] and confers a high risk of developing heart failure [22,23,24]. Heart failure is a significant long-term cardiac complication after viral infections [19,21].

### 1.2. SARS-CoV-2 and Thrombotic Events

COVID-19 is associated with coagulation abnormalities, resulting in venous and arterial thromboembolism [25]. Although the exact incidence is unclear, this includes myocardial infarction, in-stent thrombosis, and sudden left ventricular dysfunction [26]. Thrombotic events are highly variable (17–85%) in COVID-19 patients [27,28,29], and approximately 20% of COVID-19 patients develop venous thromboembolism [30]. In addition, peripheral arterial thromboembolism has been noticed in young COVID-19 patients without prior risk factors, with acute thrombosis involving the aorta presenting as acute limb ischemia [31]. In this Issue, a study by Xue et al. used Syrian hamsters for SARS-CoV-2 infection to advance the knowledge of preclinical studies on an animal model. This study described hematological abnormalities, early cardiopulmonary failure, and early thrombus formation, suggesting a similar level of pathology observed in the acute stages of SARS-CoV-2 infection in human subjects and offers platforms for evaluating the therapeutics of disease pathology [32].

### 1.3. SARS-CoV-2 and Multisystem Inflammatory Syndrome in Children (MIS-C)

Compared to adults, children are less susceptible to COVID-19 and generally have very mild clinical symptoms. A case series reported by a pediatric intensive care unit in the UK provides evidence of hyperinflammatory syndrome with features of Kawasaki disease in eight children, of which five tested positive for SARS-CoV-2 [33]. Furthermore, Verdoni et al. suggested a possible association between a high incidence of a severe form of Kawasaki disease and SARS-CoV-2, noting a 30-fold increase in the incidence of Kawasaki-like disease among children during the peak of the pandemic [34]. In this Issue, Fabi et al. reported that Multisystem Inflammatory Syndrome in Children (MIS-C) increased during the COVID-19 pandemic, with features that partially overlap with Kawasaki Disease (KD). Their cross-sectional study reported an increased level of IL-8 in MIS-C patients, who responded very rapidly to immunomodulatory treatment [35].

### 1.4. SARS-CoV-2 Vaccination and Cardiac Complications

Vaccination strategies against SARS-CoV-2 have been found to be effective in reducing infection. However, some vaccines, particularly mRNA-based vaccines, have been associated with multiple side effects, such as myocarditis/pericarditis. Several systematic reviews and meta-analyses have suggested an increased risk of myocarditis/pericarditis after COVID-19 vaccination [36,37,38]. In this Issue, Parra-Lucares et al. reviewed the current literature on vaccination-related cardiac involvement and proposed a pathophysiological mechanism for vaccine-induced myocarditis/pericarditis. They also propose that similarity between viral spike protein and autoantigen generation of autoantibodies may occur [39].

## 2. Conclusions

SARS-CoV-2 infection not only infects the lungs but also affects the extrapulmonary organs, such as the heart, which may result in long-term health conditions. This Special Issue highlighted the cardiac involvement after SARS-CoV-2 infection as well as vaccination and discussed the mechanism of heart–viral interaction.
